# Sex disparities in attitudes towards intimate partner violence against women in sub-Saharan Africa: a socio-ecological analysis

**DOI:** 10.1186/1471-2458-10-223

**Published:** 2010-04-29

**Authors:** Olalekan A Uthman, Stephen Lawoko, Tahereh Moradi

**Affiliations:** 1 Department of Public Health Sciences, Division of Social Medicine, Epidemiology, Karolinska Institutet, Stockholm, Sweden; 2 Department of Public Health & Biostatistics, University of Birmingham, Birmingham, UK; 3 Center for Evidence-Based Global Health, Ilorin, Kwara State, Nigeria; 4 Department of Environmental Medicine, Division of Epidemiology, Karolinska Institutet, Stockholm, Sweden

## Abstract

**Background:**

Attitudes towards intimate partner violence against women (IPVAW) has been suggested as one of the prominent predictor of IPVAW. In this study, we take a step back from individual-level variables and examine relationship between societal-level measures and sex differences in attitudes towards IPVAW.

**Methods:**

We used meta-analytic procedure to synthesize the results of most recent data sets available from Demographic and Health Survey (DHS) of 17 countries in sub-Saharan Africa conducted between 2003 and 2007. Pooled odds ratio (OR) and 95% confidence intervals (CI) were computed for all countries. Test of heterogeneity, sensitivity analysis, and meta-regression were also carried out.

**Results:**

Women were twice as likely to justify wife beating than men (pooled OR = 1.97; 95% CI 1.53- 2.53) with statistically significant heterogeneity. The magnitude in sex disparities in attitudes towards IPVAW increased with increasing percentage of men practicing polygamy in each country. Furthermore, magnitude in sex disparities in attitudes towards IPVAW decreased monotonically with increasing adult male and female literacy rate, gender development index, gross domestic product and human development index.

**Conclusion:**

This meta-analysis has provided evidence that women were more likely to justify IPVAW than men in sub-Saharan Africa. Our results revealed that country's socio-economic factors may be associated with sex differential in attitudes towards IPVAW.

## Background

Intimate partner violence against women (IPVAW) is present in almost all societies [1,2] and is associated with considerable mortality [1,3]. IPVAW has been linked to numerous kinds of immediate and long term physical and psychological injury in women[4]. IPVAW is integrally linked to ideas of male superiority over women[5]. These are manifest in different ways in different societies. Violence is one way to create and enforce gender hierarchy and punish transgression[6], to resolve relationship conflict and to seek resolution of crisis of masculinity by providing a sense of power[6]. Different factors influence the status of women and men in a society and thus, influence these processes[6]. These factors include social and demographic characteristics of the women and men, their economic circumstances, and the characteristics of their relationship [7-13]. Among other potential risk factors, attitude towards IPVAW has been suggested as one prominent predictors of IPVAW [14-16]. Attitudes that support IPVAW may be an indication of deeper malaise in the society[17]. High acceptance of IPVAW may suggest high levels of acceptance of violence to resolve any conflict and acceptance of violence as an instrument to retribution [17]. This may in turn suggest that IPVAW may be more common in these societies[17].

Few studies have examined gender differences in attitudes towards IPVAW in sub-Saharan Africa (SSA) [16,18,19]. Thus, there is an important gap in documenting and explaining sex differences in attitudes towards IPVAW. Without objective information about the current patterns and cross-country perspective on men's and women's attitudes toward IPVAW, it is difficult to plan meaningful public health programs to prevent IPVAW. Violence places a serious health burden on women and their children specially through its connection to the rising tide of HIV [20,21]. Therefore, the aim of this study was to explore gender differences in attitudes toward IPVAW in SSA and to examine societal level factors associated with it.

## Methods

### Data

This meta-analysis used data from Demographic and Health Surveys (DHS) conducted between 2003 and 2007 in sub-Saharan Africa available as of November 2008. DHS surveys were implemented by respective national institutions and ICF Macro International Inc. with financial support from the US Agency for International Development (USAID). Methods and data collection procedures have been published elsewhere [22]. Briefly, DHS data are nationally representative, cross-sectional, household sample surveys with large sample sizes, typically between 5,000 and 15,000 households. The sampling design typically involves selecting and interviewing separately nationally representative probability samples of women aged 15-49 years and men aged 15-59 years based on multi-stage cluster sampling, using strata for rural and urban areas and for different regions of the countries. A standardized questionnaire was administered by interviewers to participants in each country. The survey's questionnaires[23] were similar across countries yielding inter-country comparable data. Only countries with available data on attitudes towards IPVAW were included in this study. This resulted in inclusion of the following 17 participating countries in DHS: Benin, Burkina Faso, Ethiopia, Ghana, Kenya, Lesotho, Liberia, Madagascar, Malawi, Mozambique, Namibia, Nigeria, Rwanda, Swaziland, Tanzania, Uganda and Zimbabwe.

### Variables

#### Outcome variable

To assess the degree of acceptance of wife-beating by women and men, respondents were asked the following question: "Sometimes a husband is annoyed or angered by things which his wife does. In your opinion, is a husband justified in hitting or beating his wife in the following situations?" The five scenarios presented to the respondents for their opinions were: "if wife burns the food," "if wife argues with the husband," "if wife goes out without informing the husband," "if wife neglects the children," and "if the wife refuses to have sexual relations with the husband". Information was collected from all women and men irrespective of their marital status. A binary outcome variable for acceptance of wife-beating was created and coded as '0' if the respondent did not agree with any of the above mentioned five scenarios or did not have any opinion on the issue and coded as '1' if the respondent agreed with at least one scenario.

#### Country-level variable

We gathered country-level data matched within the same time frame when DHS were conducted from the reports published by the United Nations Development Programs[24] and World Bank[25]. The country-level characteristics included in this study were percent of men practicing polygamy, gross domestic product per capita, adult male and female literacy rate, gender-related development index (GDI) [26,27], and human development index (HDI) [26,28]. HDI is a composite index that measures a country's average achievements in three basic aspects of human development: health, knowledge and a decent standard of living. GDI measures achievement in the same basic capabilities as the HDI does, but takes note of inequality in achievement between women and men. The methodology used imposes a penalty for inequality such as falling the GDI when the achievement levels of both women and men in a country go down or when the disparity between their achievements increases. The greater the gender disparity in basic capabilities, the lower a country's GDI compared with its HDI. The GDI is simply the HDI discounted, or adjusted downwards, for gender inequality. To provide results that were more readily interpretable in the policy arena, we divided country-level variable into low, medium and high categories based on tiers.

### Statistical analyses

#### Meta-analysis

We calculated Odds ratios (OR) for the association between sex of the respondent and acceptance of IPVAW for each country. We used DerSimonian-Laird method (random-effects model)[29] to calculate pooled OR across countries. We evaluated the homogeneity of the results through Cochran's Q test [30]. The quantity *I*^2 ^describes the percentage variation across studies that have heterogeneity [31,32]. Negative values of *I*^2 ^were adjusted to zero (no heterogeneity) to give an *I*^2 ^between 0 and 100%, where larger values show increasing heterogeneity. We performed leave-one-country-out sensitivity analysis to determine the stability of the results. This analysis evaluated the influence of individual studies by estimating the weighted average OR in the absence of each country.

#### Meta-regression analysis

We investigated the impact of various country characteristics on OR estimates through an inverse-weighted linear meta-regression analysis. The independent variable was the natural logarithm of the OR. The explanatory factors included the country characteristics (listed above), sample size, sub-region and the calendar year of the survey. This analysis accounted for aspects such as effect modifications of the explanatory factors by performing univariable linear regression analyses for each factor. All tests were two sided and *p *< 0.05 was considered significant. Stata 10 (Stata Corp, College Station, TX, USA) software was used for analysis.

### Ethical consideration

This study was based on an analysis of existing survey data with all identifier information removed. The survey was approved by the Ethics Committee of the ICF Macro at Calverton in the USA and by the National Ethics Committees in their respective countries. All study participants gave informed consent before participation and all information was collected confidentially.

## Results

**Figure 1:**
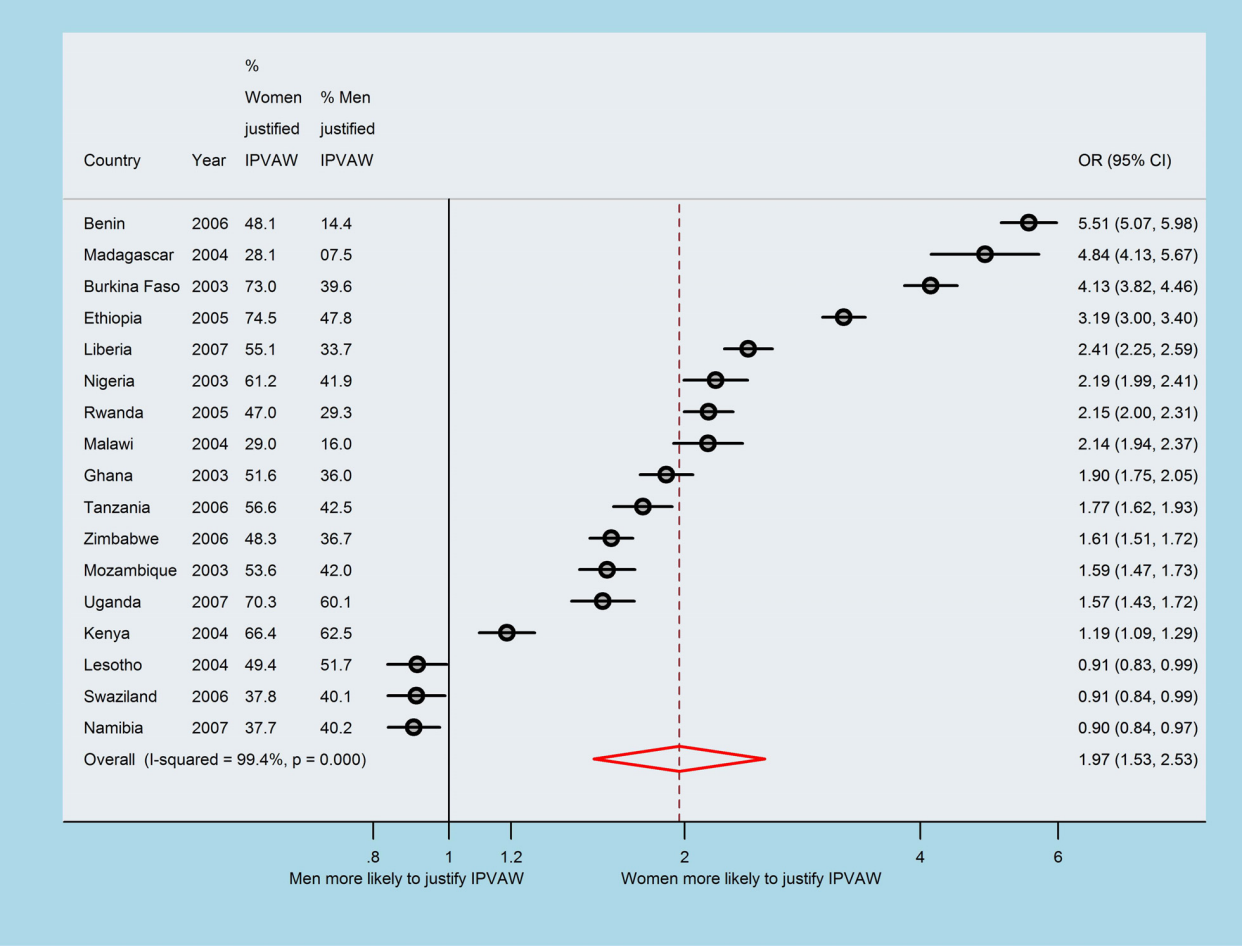
**Forest plot of weighted gender-difference in attitude toward intimate partner violence against women of 17 countries in sub-Saharan Africa**.

**Figure 2:**
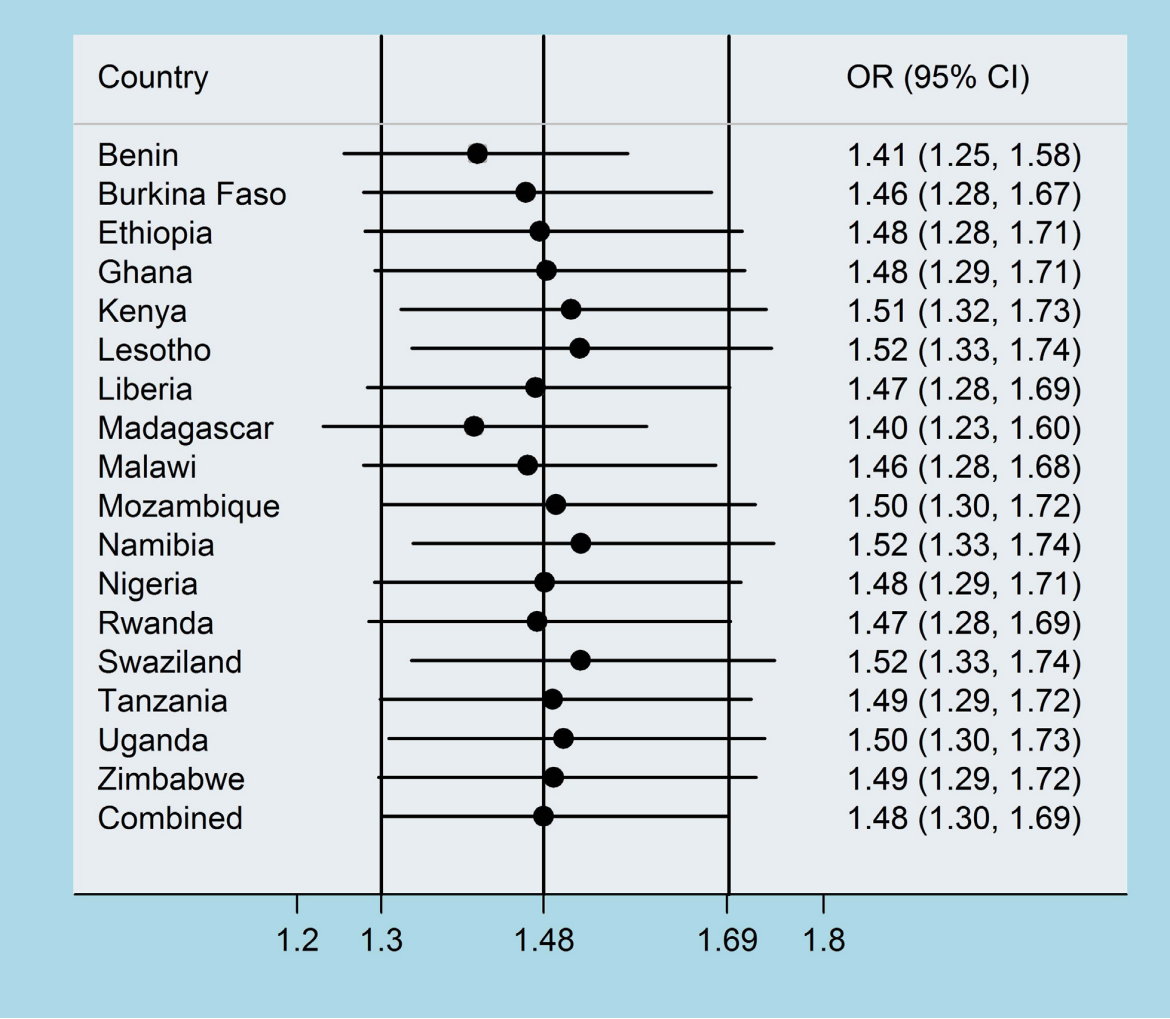
**Plot indicating the influence of each country on the overall pooled result- "leave-one-country-out" sensitivity analysis**.

Table 1 shows years of data collection, and sample sizes by selected demographic and economic diversity across 17 countries in sub-Saharan Africa (SSA). All the 17 countries are low-income countries. As for gross domestic product (GDP) per capita, Swaziland and Namibia emerged as the most affluent country with values higher than US$2000, whilst by contrast Ethiopia, Malawi, and Rwanda were the most deprived with values less than US$250. The adult female literacy rate ranged from as low as 17% in Burkina Faso to as much as 90% in Lesotho. The adult male literacy rate ranged from 31% in Burkina Faso to as much as 93% in Zimbabwe. The percentage of men with more than one wife ranged from about 3% in Liberia and Madagascar to 33% in Nigeria. Seven countries had low human development index (HDI) and ten countries had medium HDI. As shown in Figure 1, the percentage of women who justified IPVAW ranged from 28% in Madagascar to as much as 74% in Ethiopia. The percentage of men who justified IPVAW ranged from 8% in Madagascar to 62% in Kenya. Figure 1 also illustrate the odds ratio (OR) and 95% confidence interval (CI) from individual countries and pooled result. Except for Lesotho, Swaziland and Kenya, women were consistently more likely to justify IPVAW than men in most of the countries studied than men (Figure 1). The calculated pooled effect estimates were identical, assuming either a fixed- or a random-effects model (OR = 1.98; 95% CI 1.94 - 2.02) and (OR = 1.97; 95% CI 1.53-2.53), respectively. The Cochran's Q test (Q = 2617.72; *p *= 0.001) and the corresponding *I*^2 ^(99%) indicated statistically significant heterogeneity. In the leave-one-country-out sensitivity analysis the CIs did not change materially with exclusion of any of the countries, which remains within 95% confidence interval of the overall estimate for all countries (Figure 2). This analysis confirmed the stability of the results.

**Table 1:** Selected social, economic, and demographic characteristics of 17 countries in Sub-Saharan Africa by year of survey.

Country	Year	Sample size		Polygamy (%)	GDP per capita		Adult literacy rate		GDI	HDI
						
		Men	Women		Value (US$ 2005)	Growth rate (1990 - 2005)	Men	Women		
**Benin**	2006	6000	18000	11.6	508	1.4	47.9	23.3	0.422	Low
**Burkina Faso**	2003	3605	12477	18.9	391	1.3	31.4	16.6	0.364	Low
**Ethiopia**	2005	6033	14070	5	157	1.5	50	22.8	0.393	Low
**Ghana**	2003	5015	5691	13.8	485	2	66.4	49.8	0.549	Medium
**Kenya**	2003	3578	8195	4.6	547	-0.1	77.7	70.2	0.521	Medium
**Lesotho**	2004	2797	7095	na	808	2.3	73.7	90.3	0.541	Medium
**Liberia**	2007	6009	7092	3	167	2.3	58.3	45.7	Na	na
**Madagascar**	2004	2432	7949	2.6	271	-0.7	76.5	65.3	0.53	Medium
**Malawi**	2004	3261	11698	3.2	161	1	74.9	54	0.432	Low
**Mozambique**	2003	2900	12418	7.4	335	4.3	54.8	25	0.373	Low
**Namibia**	2007	3915	9804	1.1	3016	1.4	86.8	83.5	0.645	Medium
**Nigeria**	2003	2346	7620	33.3	752	0.8	78.2	60.1	0.456	Low
**Rwanda**	2005	4820	11321	10.2	238	0.1	71.4	59.8	0.45	Low
**Swaziland**	2006	4156	4987	6.2	2414	0.2	80.9	78.3	0.529	Medium
**Tanzania**	2004	2635	10329	4.9	316	1.7	77.5	62.2	0.464	Low
**Uganda**	2006	2503	8531	7.6	303	3.2	76.8	57.7	0.501	Medium
**Zimbabwe**	2006*	7175	8907	4.2	259	-2.1	92.7	86.2	0.505	Medium

(Source: ^†^Demographic and Health Surveys of the respective countries; ^‡^UNDP Human Development Report)

GDP: gross domestic product, HDI: human development index, GDI: gender-related development index; na: not available

Table 2 shows the univariable inverse-weighted linear meta-regression results. On meta-regression, the geographical sub-region where the study was conducted was a significant predictor of heterogeneity in sex differences in attitudes toward IPVAW. Both the sample size and the calendar year when the survey was conducted was significantly associated with odds of women justifying IPVAW than men. The odds of women justifying IPVAW than men increased with increasing percentage of men practicing polygamy in each country. Furthermore, the odds of women justifying IPVAW than men decreased monotonically with increasing adult male and female literacy rate, gender development index, gross domestic product and human development index.

**Table 2:** Univariable meta-regression of attitudes towards intimate partner violence using study and country-specific characteristics as explanatory factors

*Variable*	Ratio of OR (95% CI)
**Study characteristics**	
Calendar year	
2003 -- 2004	1 (reference)
2005 -- 2006	1.42 (1.36, 1.49)
2007	0.62 (0.58, 0.65)
Sample size (per 10,000)	
9000 -- 11000	1 (reference)
11000 -- 15900	0.98 (0.93, 1.03)
>16000	4.15 (3.95, 4.36)
Country-covariates*	
Polygamous (%)	
Low	1 (reference)
Average	1.26 (1.20, 1.32)
High	3.96 (3.77, 4.16)
Gross domestic product per capita	
Low	1 (reference)
Average	1.47 (1.40, 1.54)
High	0.28 (0.27, 0.30)
Adult male literacy rate (%)	
Low	1 (reference)
Average	0.30 (0.29, 0.32)
High	0.17 (0.16, 0.18)
Female adult literacy rate (%)	
Low	1 (reference)
Average	0.39 (0.37, 0.40)
High	0.15 (0.14, 0.16)
Gender development index	
Low	1 (reference)
Average	0.22 (0.21, 0.23)
High	0.17 (0.16, 0.18)
Human development index	
Low	1 (reference)
Medium	0.23 (0.22, 0.24)
Region	
Southern Africa	1 (reference)
Eastern Africa	0.32 (0.30, 0.33)
Western Africa	0.10 (0.09, 0.11)

(**Source**:* authors construct based on data from UNDP statistics)

## Discussion

This first known meta-analysis on sex differences in attitudes toward IPVAW brought together evidence from 17 countries in sub-Saharan Africa. We found that women are more likely to justify IPVAW than men in most of the countries studied. This study confirm the findings of previous study that examined this association [18]. Sub-Saharan African countries are ethnically and religiously diverse with economic development and education levels that vary widely across these countries. As would be expected, we found highly significant heterogeneity in sex differences in attitudes towards IPVAW across countries. However, it is not assumed that the beliefs in the women lead to their abuse and battering by men or that men who accept IPVAW are more likely to be wife abusers[18]. However, women who maintain these beliefs may be at a greater risk of continuous abuse than those who do not[18]. In addition, women's susceptibility to IPVAW is shown to be greatest in societies where the use of violence is a socially accepted norm[5] which leads to women's inactivity in opposing violence against themselves [33]. Similarly, high normative acceptance among men may make it difficult for them to realise the abuse they perpetuate[18]. Fear of violence for refusing sexual relations may have important implications for the efforts to stall progress of HIV/AIDS epidemic in this region[18]. Women condemnation of this behaviour may, therefore, be an important element in changing it

Meta-regression analyses suggests that societal level variables may be important factors associated with the observed sex differences in attitudes towards IPVAW. We found that the odds of women justifying IPVAW more than men increased with increasing country polygamy rate and decreased with increasing adult male and female literacy rate. Similarly, the likelihood of women justifying IPVAW more than men decreased monotonically with country's increasing economic status, gender development index, and human development index. These findings have some policy and programme implications. At country-level increasing adult literacy and employment rates may come a long way in modifying attitudes towards IPVAW. Given the societal factors that shape the behaviour of communities and individuals, we believe that structural interventions hold great promise for significant achievements in the prevention of IPVAW[34]. The structural public health intervention could include: fostering gender equality and women's empowerment and integrating IPVAW prevention into other programme areas. Direct concerted efforts from the government, non-governmental organisations and enlightened men and women within the society are necessary to raise awareness about the issue and question the social norms[18].

The findings of our comparative analysis should be interpreted in the context of both intrinsic limitations of meta-analysis, and in the context of our own study-specific (subject matter) limitations. In meta-analysis, the traditional unit of analysis is each study (country in our case), thus, compared with multilevel analysis with individual-level data, the power to detect a difference in aggregate or to identify explanatory variables by meta-regression is greatly diminished. As with all ecological studies, the findings of this study cannot be considered conclusive because of the cross-sectional and ecological design and the possibility of ecological fallacy. Thus, caution should be exercised in the attribution of a casual relationship and the direction of relationship observed in the study. Another limitation is that the meta-regression analysis is based on univariable analysis, due to small sample size (number of countries included), the study could not control for potential confounders simultaneously using multivariable meta-regression analysis. Despite these limitations, the study strengths are significant. It is a large, population-based study with national coverage. It is increasingly recognised that, even when studying individual level risk factors, population level studies play an essential part in defining the most important public health problems to be tackled, and in generating hypotheses as to their potential causes[35]. An important aspect of any meta-analysis is to conduct a thorough search of published studies which should then be included in the pooled estimate[36]. We took a different approach in this study. While the methods of synthesizing data from various studies were formulated in the context of epidemiology and clinical trials research, these methods are applicable, with appropriate modification, to health research surveys as well[37]. Meta-analysis involving health survey may seem odd since they have not often meta-analysed. However, the effect size are straightforward if two conditions are met[38]. First, all the findings must involve the same variable operationalized in the same way or in sufficiently similar ways that the numerical values have comparable meaning across surveys [38]. Second, it must be possible to define effect size statistics that represents the information of interest and to determine the standard error associated with that statistic[38].

We directly analysed substantial number of public domain data sets instead of using results from published studies. This approach, we believe, brings with it two considerable advantages. First, not all published studies include the same or even comparable variables in their analyses. Taking raw data from DHS allow us to use the same variables and most comparable items for attitudes toward IPVAW. Second, there are many more survey data sets than there are published studies. We therefore, achieve a much greater coverage of the population of effect sizes and mitigate the 'publication bias' that increases the probability of Type I errors[39]. This is a problem that makes meta-analysis prone to overestimating effect sizes where the data are collected solely from published work[40].

## Conclusion

This meta-analysis has provided evidence that women were more likely to justify IPVAW than men in sub-Saharan Africa. We found that the better the country economic status, adult female and male literacy rate, gender development index, human development index the higher the sex disparities in attitudes toward IPVAW. However, it is important to note that there was high heterogeneity in sex differences in attitudes towards IPVAW across countries. Thus, suggesting that multifaceted geographically differentiated intervention may represent a potentially effective approach for addressing issues related to IPVAW in sub-Saharan Africa and policies have to be tailored to country-specific conditions.

## Competing interests

The authors declare that they have no competing interests.

## Authors' contributions

OAU, SL and TM were involved in the conception of the study. OAU carried out data extraction. OAU conducted statistical analysis under supervision of SL and TM. OAU drafted the paper with contributions from the co-authors. All authors read and approved the final manuscript.

## Pre-publication history

The pre-publication history for this paper can be accessed here:

http://www.biomedcentral.com/1471-2458/10/223/prepub
